# Is an 8-Week Regimen of Nordic Walking Training Sufficient to Benefit Cognitive Performance in Healthy Older Adults? A Pilot Study

**DOI:** 10.3390/jcm13051235

**Published:** 2024-02-21

**Authors:** Marta Maria Torre, Clelia Carrubba, Antoine Langeard, Nicolas Hugues, Jérôme Laurin, Jean-Jacques Temprado

**Affiliations:** 1 Institut des Sciences du Mouvement (ISM), Aix-Marseille Université, CNRS, 13288 Marseille, France; clelia.carrubba@univ-amu.fr (C.C.); nicolas.hugues@univ-amu.fr (N.H.);; 2 Institut National de la Santé et de la Recherche Médicale (INSERM), Mobilités: Vieillissement, Pathologie, Santé (COMETE), Université Caen Normandie, CHU, 14032 Caen, France; antoine.langeard@unicaen.fr; 3 Institut de Neurobiologie de la Méditerranée (INMED), Institut National de la Santé et de la Recherche Médicale (INSERM), Aix-Marseille Université, 13273 Marseille, France; jerome.laurin@univ-amu.fr

**Keywords:** aging, Nordic walking, training, cognition

## Abstract

Nordic walking requires the association of walking and coordination of limbs while orienteering in a natural environment. It has been shown to improve functional capacities more than normal walking. However, its cognitive benefits are less clear. The main hypothesis was that this training improves visuospatial capacities and inhibition functions. A total of 14 healthy older adults were included. The training was performed in three sessions of 75 min a week for 8 weeks. Pre-, intermediate, and post-tests were carried out. Cognitive functions including global cognition (MoCA), executive functions (Color–Word Stroop test), speed of information processing, switching capacities (Trail Making Test A and B), and visuospatial capacities (Rey Complex Figure Copy Task) were assessed. Motor functions including balance control (Unipedal Balance Test), functional mobility (Timed Up and Go), hamstring flexibility (Chair Sit and Reach test), and motor coordination (Four-Square Stepping Test) were evaluated. Physical function, including lower limb strength (Timed Sit-To-Stand) and cardiovascular capacities (Incremental Shuttle Walking Test), was measured. Cardiovascular capacity, strength of lower limbs, and motor coordination were positively affected by training. With respect to cognition, training improved visuospatial capacities, while switching capacities, information processing speed, and executive functions did not improve. A possible explanation is that they needed a longer program duration to show benefits. However, analyses of responders suggested that NW positively affected cognitive functioning in a subset of participants. Eight weeks of NW training produced physical, motor, and cognitive improvements. A longer training duration could be necessary to extend the benefits to executive functions in all participants.

## 1. Introduction

The burden of aging-related physical, motor, and cognitive declines is even more marked in sedentary individuals [1]. Accordingly, delaying or (even partially) compensating for those functional capacity alterations is critical for the growing number of older adults in the general population. In particular, improving cognitive functions (attention, inhibition, visuospatial capacities, etc.) is important because they strongly determine behavioral adaptability in everyday life situations and complex movement tasks [2,3,4,5]. It is widely accepted that both endurance and muscular resistance training, when performed separately or in association, effectively counteract the effects of aging on cognitive capacities [6,7,8,9]. More recently, it has been suggested (and, in some cases, demonstrated) that training programs that integrate physical, motor, and cognitive stimulations could be more effective than separate training programs in improving brain plasticity and cognition in older adults (for details, see [10]). However, which type of “conventional” physical activity can ensure an effective combination of cognitive, motor, muscular, and cardiovascular stimulation is still debatable. 

Nordic walking (NW) can be considered a good candidate in this respect, Indeed, it is not only it is a safe and much-appreciated activity for older adults; it is also more beneficial than normal walking in improving functional capacities, particularly maximal oxygen consumption [11,12]. Moreover, it has been suggested that NW could be more appropriate than most conventional activities for improving cognition because it requires a combination of aerobic effort, complex motor skills, and cognitive stimulation [13]. For this reason, according to the Adaptive Capacity Model (ACM) [14], it should be effective in improving brain plasticity and cognitive performance [10,14]. In particular, in addition to stimulating brain plasticity through an increase in neurotrophic factors’ concentrations, Nordic walking can improve cognitive functions by requiring the coordination of upper and lower limbs while orienteering in a natural environment, as in dual-task training [13]. However, despite the potential interest in NW for improving brain plasticity and cognition in healthy older adults, very few studies have addressed this issue. Thus, though they showed significant benefits of NW on cognition [13,15,16,17,18,19] (but see [20] for more inconsistent results) as pointed out by Nemoto et al. [13], additional cognitive testing should be conducted to determine whether and under which conditions cognitive functions may be enhanced by NW training. 

In particular, two main questions deserve to be addressed in this respect. First, what is the minimum training duration for Nordic walking to be effective in improving cognitive capacities? Indeed, training duration is considered a moderator of the effects of training in any type of activity (i.e., physical, motor, or cognitive). Unfortunately, knowledge of the mechanisms at work in different types of activity does not make it possible to set a gold standard for each activity. Thus, the determination of the most effective durations is carried out by ‘trial and error’. For endurance activities (such as NW), the most used and ultimately commonly adopted duration is 12 weeks. The question arises as to whether a shorter duration may be effective, particularly in training that combines physical, motor, and cognitive stimulation (such as NW) for which effective training durations may differ. For example, the duration required for cognitive training is generally shorter than that required for physical training, even if this question has not been theorized in the literature. Most studies have shown improvements in physical, motor, and cognitive functions with training programs lasting 12 weeks or more [16,17,18]. Other studies have also reported the beneficial effects of shorter NW training programs (i.e., 8–9 weeks) on physical and motor abilities [12,21,22], but cognitive performance was not measured in these works. Thus, it remains unknown whether short NW training programs (even 4 weeks) can benefit cognition, together with physical and motor fitness. For this reason, a question arises regarding the effectiveness of training for 8 weeks or even 4 weeks as measured in the present study. The answer to this question could have important practical consequences for the aging population, the majority of whom are not very active, such that the prospect of having to engage in physical activities for a long time to obtain significant benefits can be discouraging.

A second question concerns which cognitive functions are positively affected by repetitive practice of Nordic walking. In a previous study, we showed that executive functions and information processing speed were enhanced after an NW training program [19], but a global picture of the cognitive functions that can be improved by NW is still lacking because the cognitive functions measured and the tests used are frequently different from one study to the other. 

The current study addressed these issues by investigating the time-dependent effect of an NW training program on physical, motor, and cognitive functions in healthy older adults after 4 and 8 weeks, respectively (i.e., 12 and 24 sessions). Our main hypothesis was that, in addition to improving physical and motor capacities, NW training should enhance cognitive functions, even after a short-duration training program (i.e., 4 and 8 weeks), though perhaps with different time profiles of performance over the two subparts of the training program (i.e., the first and second 4-week periods). This hypothesis was based on the premise that NW, as a combined training intervention, not only stimulates the release of neurotrophic factors in the bloodstream, thereby facilitating brain plasticity and cognition but could also trigger a “guidance” effect thanks to the cognitive load involved in the complex movements performed during NW [13]. As a result of combined stimulations, improvements in cognition could be observed even after an intervention of short duration, as we demonstrated for motor coordination training in a previous paper [5]. This hypothesis is also consistent with Diamond and Ling’s [23] statement that the effects of endurance exercise on cognition were more marked when supported by complex motor skills (see also [2] for a convergent point of view). 

## 2. Material and Methods

### 2.1. Participants

Participants were recruited among community-dwelling independent-living older adults based in Marseille (France) based on a preexisting list of volunteers recruited through a notice in the local newspaper. After contacting the project manager, either through phone calls or e-mails, volunteers were screened for the first time for inclusion. Participants were considered eligible if (i) they were between 65 and 80 years of age, (ii) they had not taken part in any sports activity or training program in a club or an association during the last 3 months, and (iii) they had no previous experience in Nordic walking. The exclusion criteria were as follows: (i) a score below 26 on the Mini-Mental State Examination (MMSE) [24], (ii) cognitive impairment or language disorders that would preclude understanding instructions, (iii) an uncontrolled psychiatric or cardiovascular condition, (iv) uncorrected vision and/or hearing, and (v) psychotropic or bradycardic therapy. 

Participants were given practical information related to the training programs (location, schedule, etc.), as well as their rights, benefits, and potential risks. To volunteer, they were asked to sign an informed consent. After consenting, participants underwent an accurate and detailed set of psychiatric, psychological, physical, and medical screenings by a geriatrician to be confirmed eligible to participate. 

**Figure 1:**
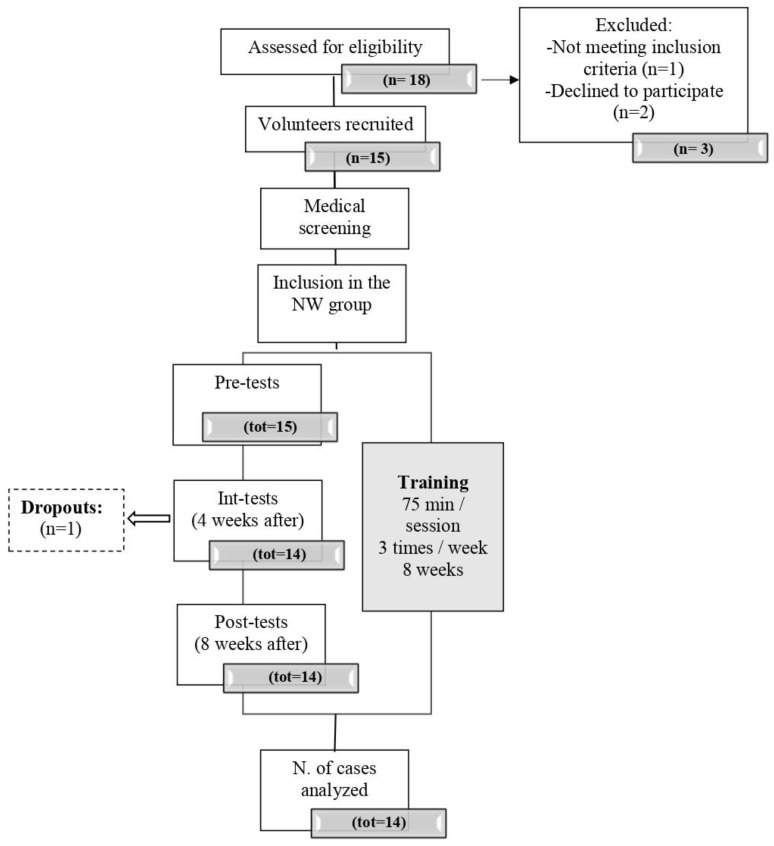
Flowchart of participant recruitment and admission.

Their capacity to perform moderate to vigorous effort (60–70% of maximum) was assessed through a submaximal stepping test conducted at the Clinique Provençale de Médecine du Sport of the Saint Marguerite hospital in Marseille and supervised by a cardiologist. The specialist adapted the test protocol if the subject reported previous cardiovascular problems. The sample’s baseline characteristics are shown in Table 1, and the flowchart is shown in Figure 1.

### 2.2. Sample Size

The sample size calculation was performed using G*Power 3.1.9.7 (Kiel, Germany) [25]. To detect relevant differences in the changes from the pre-test to the intermediate test and the post-test, respectively, we calculated a sample size sufficient to detect medium effect sizes. Therefore, f = 0.35, =0.05, and *p* = 0.80, were chosen to favor clinically significant effect sizes. To test the main hypotheses of the present study, 15 participants were required. A total of 15 volunteers were initially recruited to participate, but one dropped out immediately before the intermediate tests/assessments, and 14 healthy older adults completed the study.

### 2.3. Study Design and Training Intervention

The protocol was conducted according to the Declaration of Helsinki and approved by the French National Ethics Committee (CPP IDF10 no. 2019-A03263-54; May 2020).

The 8-week Nordic walking training intervention took place in a natural park, with rugged terrain offering multiple ways of different lengths to modulate the difficulty and, consequently, the orientation processes, the required level of endurance effort, and the complexity of gait and postural control. 

The program consisted of three sessions of 75 min each per week (i.e., 24 h in total). Each training session was organized as follows: (i) a 15 min warm-up phase including exercises for muscular activation and motor coordination; (ii) a 50 min core training phase, and (iii) a 10 min cool-down phase with stretching exercises to restore baseline HR values. Participants were provided with heart rate monitors (Decathlon HR300™, Decathlon, Nord-Pas-de-Calais, France) and were instructed on how to record their heart rates, at rest and during NW sessions. The heart rate monitor allowed the coach and the scientific team to ensure that their mean heart rates during each session were close to 70–80% of their estimated HRmax, calculated with the conventional formula of Tanaka et al. (208 − 0.7 × age) [26]. 

The training intervention was supervised by an experienced coach, officially licensed in a multisport club (Stade Marseillais Université Club), who led training sessions of adapted physical activity for older adults. The coach volunteered to participate in the experiment. The participants were informed that their data recorded during assessments would be anonymized and analyzed to determine the effectiveness of the training program. 

The scientific team (i.e., the authors) had several work sessions with the coach before the training intervention to define the different parts of the sessions and their contents. They allowed to fix the training strategy according to general principles. In particular, the coach was asked to design exercises requiring spatial orientation in the natural park where the sessions took place. Additionally, he was asked to include, during the warm-ups of the sessions, technical exercises to teach the participants how to coordinate the upper limbs with walking and how to use the sticks. Furthermore, the coach had to continuously increase the intensity of endurance effort and step control on cluttered terrain, over the 8 weeks, individualizing the request, as in our previous study [18].

Finally, a member of the scientific team observed all the sessions to check whether the instructions were respected by both the coach and the participants. The reported observations were discussed after each session with the coach and the team, which allowed us to adapt the contents of the sessions over the training period, as a function of the behaviors produced by the different participants.

### 2.4. Assessment Procedure

Pre-tests were carried out one week before the training period started. Intermediate tests (i.e., after 4 weeks) and post-tests (i.e., after 8 weeks) were also carried out. Cognitive, physical, and motor performance were assessed at the Institute of Movement Science (ISM) (Marseille, France). All test sessions were organized and managed by several members of the scientific team with the help of students of the Faculty of Sport Sciences in Marseille, specializing in Adapted Physical Activity. The coach did not participate in the assessment sessions, and he was not informed about the outcomes of the assessments before the end of the training program. 

#### 2.4.1. Cognitive Assessments

Global cognitive performance was tested with the Montreal Cognitive Assessment (MoCA). This test is considered highly sensitive to detecting mild cognitive impairment (MCI). To avoid test–retest effects, the three different versions of this test were randomly assigned to the pre-test, intermediate test, and post-test sessions for each participant [27]. 

Executive functions were assessed through three different tests dedicated to (i) inhibition function, (ii) information processing speed and cognitive switching, and (iii) visuospatial capacities, respectively.

Inhibition processes were tested with a computerized version of the Color–Word Stroop test (CWST). Participants were seated in front of a computer screen, and a special keyboard with only four letters [R for rouge (red), V for vert (green), J for jaune (yellow), and B for bleu (blue)]. Different (French) words appeared on the screen, one by one, written in one of the previously listed colors. Participants had to indicate the color in which the word was written by pressing, as quickly as possible, the corresponding letter on the keyboard with the forefinger of the dominant hand while inhibiting the word’s semantics. Thus, depending on the consistency between the word’s semantics and the color, the condition was considered either congruent (C, e.g., the word “green” written in green), incongruent (I, e.g., the word “green” written in red), or neutral (N, e.g., words such as arm, leg, etc., written in one of the different colors). After a familiarization task with 9 random words, the test began, consisting of 75 words (25 C, 25 N, and 25 I). Each word remained on the screen until the response was given on the keyboard. Along with the error rate, the response time (RT), the time elapsing between the appearance of the word on the screen and the manual pressing of the key on the keyboard, was measured and averaged in each of the three conditions for each participant. 

Information processing speed and switching capacities were evaluated through the Trail Making Test (TMT) parts A and B, respectively. In part A, participants had to draw a continuous line connecting 25 encircled numbers in numerical order. Part B required participants to perform the same task, but alternating numbers and letters (e.g., 1, A, 2, B, 3, C, etc.) while respecting the numerical and alphabetical order [28]. The total time (in milliseconds) and the number of errors were recorded. 

Visuospatial capacities were tested using the Rey Complex Figure Copy Task [29] (RCFCT). It consisted of manually reproducing on a sheet a complex figure comprising 18 graphical elements placed in front of them, to the best of the participant’s ability. Self-correction was allowed. The completion time required to copy the figure was reported in milliseconds, and a score was given for each element that was reproduced correctly (maximum score = 18).

#### 2.4.2. Motor Fitness Assessments

Balance control was assessed with the Unipedal Balance Test (UBT). Participants, with eyes open, were asked to lift one of their legs, placing the foot on the ankle of the supporting leg [30]. They were instructed to keep their arms along the torso, and a mark was placed on the wall in front of each participant matching their different eye heights, to provide a fixation point. During the test, participants were requested to maintain stable balance on their preferred leg (one familiarization trial allowed them to choose it) for as long as possible (the maximum duration was 1 min). Then, two trials were performed, and the best performance was recorded. The time (in seconds) elapsing from the go signal (starting the chronometer) until balance was lost (if they lost balance and lifted their arms past hip height) was measured.

The Timed Up and Go test (TUG) assessed functional mobility. Participants sat in a chair, keeping their feet on the floor and their hands resting on their legs. At the “go” signal, they stood up, walked at their own pace, turned around a cone placed 3 m away from the chair, walked back and sat down again. The completion time was recorded, starting the stopwatch at the initial movement to rise from the chair and stopping it when they sat down again. Two trials at a self-paced walking speed and two as fast as possible were performed. The best record between the two trials of each of the two different conditions was retained [31]. 

Motor coordination was assessed using the Four-Square Stepping Test (FSST) [32]. The square was created by placing on the floor two 90 cm long Scotch tape lines, crossed at their midpoint. The four squares were enumerated with 1 in the upper left square, 2 in the upper right square, 3 in the lower right square, and 4 in the lower left square. Participants were asked to stand in square number 1, facing square number 2, and they had to step as fast as possible into each square following a sequence of 2, 3, 4, 1, 4, 3, 2, and 1, requiring them to step forward, backward, and sideways to the right and left. The instructions were as follows: “Complete the sequence as fast as possible without touching the lines of the square, both feet should touch the floor in each square while facing forward”. After a demonstration, one familiarization trial was completed to ensure the participant’s comprehension of the task. The time taken to complete the sequence (in seconds) was recorded. The stopwatch started when the first foot contacted the floor in square 2 and stopped when the last foot touched the floor in square 1. The better of two trials was retained for the analysis. If the participant failed to complete the sequence successfully, lost balance, or touched a line during the sequence, they were allowed to repeat the task. Participants were constantly supervised for safety’s sake. 

#### 2.4.3. Physical Assessments

Lower limb strength was assessed with the Timed Sit-To-Stand (STS) test [33]. The test began with the subject seated with the back straight on a chair without armrests and placed against a wall. They were then asked to place the feet on the floor, shoulder-width apart, and to cross the arms on the chest. The task was, at the signal, to fully rise and sit down to the initial seating position 10 times as fast as possible. Participants had to perform the repetitions without the help of their arms. Two repetitions were allowed before the test session, to assess and fully understand the movement. The completion time was recorded. 

The Shuttle Walking Test (SWT) was chosen to test the cardiovascular capacities [34]. It is performed on a 10-m course, marked by two cones placed 0.5 m from each endpoint; the test starts at 0.5 m/s, and the speed increases by 0.17 m/s every minute. In the present study, the protocol has been adapted by extending the test to 15 levels (originally 12) of 1 min because only healthy subjects are involved. The walking pace was set with a metronome: periodic beeps indicated when participants were meant to turn around the cone. The test ended when they were unable to pivot around the cones at the occurrence of the auditory signal on three consecutive occasions (i.e., they were >0.5 m from the cone), when they were breathless and/or fatigued, or when they showed signs of physical discomfort (symptoms such as dyspnea, dizziness, and vertigo). Verbal instructions were given by examiners to the participants when they were not synchronized with the required pace, allowing them to adjust their pace as needed. During the SWT, the total distance (TD) covered was recorded for the analysis, together with the baseline HR (HHR, before the beginning of the test) and post-exercise HRmax. 

### 2.5. Attendance Rate and Training Load Assessment

Adherence to training programs was measured by calculating the attendance rate (in % of the number of sessions) over the first and second parts of training (i.e., after the first and the second periods of 4 weeks, respectively), as well as over the entire 8-week period (see Table 2). At each session, the training intensity was measured by recording the mean and maximal HR reached using a heart rate monitor. They were calculated as % of the theoretical maximal HR, which was calculated for each subject using the classic formula proposed by Tanaka et al. [26] (see [18] for a similar procedure).

### 2.6. Data Management

Selected participants were indexed with specific identification numbers so that the data collected were anonymized. Data were saved on a secured hard disk, and for each identification code, a logbook was started in which test results from baseline, intermediate, and post-intervention assessments were collected along with the documentation on each training session.

### 2.7. Data Analysis and Statistics

SPSS 28 was the program used to analyze the data (SPSS Inc., Chicago, IL, USA). All data were assessed for normality with the Shapiro–Wilk test before statistical analysis. Then, repeated-measures ANOVA, with “Time” as a within-subject condition, was carried out to test the effects of the training intervention on normally distributed dependent variables. Friedman tests were performed for the non-Gaussian data.

The analysis included the subjects who completed all the tests. The alpha level of significance was set at 0.05. The Greenhouse–Geisser correction was applied in case of violation of the sphericity assumption; effect sizes were calculated using partial eta squared (ηp2), and Newman–Keuls post hoc tests were then performed to detect differences over the three-time points. The Wilcoxon signed-rank test was performed immediately after significant Friedman tests, and the effect size was calculated using the following formula: r = Z/√N [35]. 

We analyzed the amplitude of progress, expressed in % of initial performance (Δ% = [post-test score–pre-test score)/pre-test score] * 100) in three time periods: pre–int (first 4 weeks—F4W), int–post (second 4 weeks—S4W), and pre–post (total of 8 weeks—T8W), respectively. Finally, we also counted the number of responders for the different periods (i.e., participants who progressed by more than 1%), and we calculated the corresponding amplitude of progress. No statistical analyses were carried out on these data (for a similar procedure, see [18]).

## 3. Results

### 3.1. Attendance of the Training Program

Over the twenty-four sessions planned (3 sessions × 8 weeks), all participants attended more than 80% of the training sessions (see Table 2). 

### 3.2. Training Load

Descriptive results for training load are presented in Table 3. These data reveal that the training load was within the range that was defined and targeted for the present training program (60–80% of the maximum).

### 3.3. Physical Assessments

A significant effect of time was found for the distance covered during the SWT [χ2 (N.14, df.2) = 7.51, *p* < 0.05]. Specifically, according to the Friedman test, the distance covered significantly increased from the intermediate test to the post-test (*p* < 0.05, 590 m and 668 m, respectively) but neither from the pre-test to the intermediate test (612 m and 590 m, respectively) nor from pre-test to the post-test (612 m and 668 m, respectively). Consistent with these observations, the number of responders appeared larger during S4W (64%) than during F4W (14%) and T8W (36%). Finally, on average, the participants progressed by about 10% during the training program, especially during the second 4-week period (S4W). On the other hand, when measured for responders only, the progress of the distance during the SWT was much larger, that is, 15.3% (F4W), 26.5% (S4W), and 19.1% (T8W), respectively.

Maximal HR after the SWT did not show significant differences across periods (*p* > 0.05). On the other hand, HRR significantly changed across periods [F (2,26) = 15.4, *p* < 0.05, ηp2 = 0.54]. Specifically, it decreased between the pre-test and the intermediate test (*p* < 0.05, 87.8 beats/min and 74.4 beats/min, respectively) and between the pre-test and the post-test (*p* < 0.05, 87.8 beats/min and 74.8 beats/min, respectively). No significant decrease was reported between the intermediate test and the post-test (74.4 beats/min and 74.8 beats/min). Thus, concerning HRR, participants progressed by about 14% during F4W and stabilized this progress during the 8 weeks of the training program (T8W). Notably, for HRR, the number of responders was higher during F4W (93%) and T8W (93%), while it was lower during S4W (36%). 

In the STS test, repeated-measures ANOVA reported significant decreases in execution time across periods [F (2,26) = 11.17, *p* < 0.05, ηp2 = 0.46]. Specifically, execution time decreased from the pre-test to the intermediate test (*p* < 0.05, 18.0 s and 15.5 s, respectively) and from the pre-test to the post-test (*p* < 0.05, 18.0 s and 14.6 s, respectively), but no significant difference was found between the intermediate test and the post-test (15.5 s and 14.8 s). Thus, all the participants progressed by about 16% from the pre-test to the post-test. Concomitantly, the percentage of responders was high during F4W (100%) and T8W (86%), while it was lower during S4W (64%). In addition, responders progressed by about 24% from the pre-test to the post-test.

### 3.4. Motor Assessments

For the FSST, repeated-measures ANOVA showed significant decreases in time performance over the different tests [F (2,26) = 18.54, *p* < 0.05, ηp2 = 0.59]. Post hoc comparisons showed that execution time decreased from the intermediate test to the post-test (9.1 s, and 6.9 s, respectively, *p* < 0.05) and from the pre-test to the post-test (9.6 s and 6.9 s, respectively, *p* < 0.05). No difference was observed between execution times measured during the pre-test and the intermediate test (*p* > 0.05, 9.1 s, and 9.6 s, respectively). Thus, the participants progressed by about 24% during S4W, which was also the amplitude of changes over the 8 weeks (T8W). Concomitantly, an increase in the number of responders was observed during S4W (93%) and T8W (86%) relative to F4W (57%). Responders progressed by about 33% from the pre-test to the post-test. 

Non-significant effects of periods were found for the other tests, that is, the one-leg stance test and the TUG at the preferred walking speed (*p* > 0.05). Accordingly, the numbers of responders observed in the one-leg balance test and the TUG were low. 

### 3.5. Cognitive Assessments

No significant effects of periods were observed for response times in the CWST (C, I, and N conditions), the TMT A and B, or the MoCA (*p* > 0.05). These results reflected the small number of responders observed in these tests (about 50–60%) and, as a consequence, low progress for the whole group of participants (i.e., around 4–5%). On the other hand, when calculated for the (small number of) responders in the different tests, the progress in performance was larger. For instance, a decrease of 27.8% was observed for response time in TMT A and 31% in TMT B after 8 weeks. Similarly, in the CSWT, response times decreased by about 26% (Neutral condition), 14.7% (Congruent condition), and 12.2% (Incongruent condition) after 8 weeks of training. 

On the other hand, significant effects of training periods were observed for the time to complete the Rey test [χ2 (N.14, df.2) = 7.00, *p* < 0.05]. Post hoc comparisons carried out with the Wilcoxon rank-sum test showed significant decreases in execution time from the pre-test to the intermediate test (*p* < 0.05, 155 ms, and 125 ms, respectively) and from the pre-test to the post-test (*p* < 0.05, 155 ms and 110 ms, respectively). On the other hand, no significant difference was observed between the intermediate test and the post-test (*p* > 0.05, 125 ms, and 110 ms, respectively). Concomitantly, the number of responders observed for response time in the Rey test was over 70% in all three time periods (F4W = 71%, S4W = 71%, T8W = 79%). Thus, all the participants progressed by 19% during F4W and by 30% after 8 weeks of training (T8W), while the responders progressed by about 30% and 41% during the same periods. For the performance scores, significant differences were reported between the different periods [χ2 (N.14, df.2) = 10.94, *p* < 0.05]. Post hoc comparisons showed a significant improvement in the performance scores between the pre-test and the intermediate test (*p* < 0.05, 16.5, and 17.8, respectively). In contrast, a significant decrease in performance was observed between the intermediate test and the post-test (*p* < 0.05, 17.8, and 16.8, respectively). Finally, no significant difference was observed between the pre-test and the post-test (16.5 and 16.8, respectively) (see Table 4A,B).

## 4. Discussion

The general objective of the present study was to determine whether 4 and/or 8 weeks of Nordic walking training was enough to be effective in improving cognitive capacities and, if so, to identify the most strongly affected functions. Thus, we described the temporal dynamics of progress of cognitive functions by comparing performance observed during the first and the second 4 weeks of the training periods, respectively. Accordingly, participants were tested three times on cognition (global cognitive functions, visuospatial capacities, inhibition, flexibility, and information processing speed) but also on motor and physical functions. In addition, we analyzed the individual responses of participants by counting the number of responders for each training period and calculating the amplitude of their progress as a piece of complementary information to classic statistical analyses (for a similar procedure, see [18]). Thus, we were able to compare the results of the present study with those observed on similar functions after a longer training program (e.g., 12 weeks; [16,17,18]). Additionally, we expected to add supplementary information to the studies that reported positive effects of short NW training programs (i.e., 8–9 weeks) on physical and motor capacities [12,21,22], but did not measure cognitive performance.

Theoretically, this study was motivated by the hypothesis that even a short training program in NW should have significant benefits on cognitive functions due to the combination of physical, motor, and cognitive stimulation. Indeed, Colcombe and Kramer’s pioneering studies [36,37] demonstrated that endurance training may allow enhancing cognitive and brain function in older adults [9,36,37,38,39,40,41]. Other studies showed that muscular force training also improved brain functioning and cognitive performance (e.g., [9,38]). These improvements are presumably mediated by the facilitation of brain plasticity stimulated by the release of neurotrophic factors into the bloodstream (e.g., [42,43]). These benefits can be obtained thanks to training programs based on normal walking, to the extent that walking speed is brisk enough to elicit moderate to high levels of endurance effort and muscular forces. In this respect, Nordic walking is hypothesized to have at least comparable effects on the facilitation of brain plasticity, perhaps even larger effects than normal walking. Indeed, it has been shown that NW corresponds to a higher metabolic load than natural (e.g., [22,44]) or recreational walking [45]. Accordingly, the expected added value of NW training should result from the fact that it capitalizes on both the facilitation and the “guidance” effects, thanks to the cognitive load involved both in the required complex coordinated movements [13] and in the spatial navigation mechanisms used during exercises in a natural environment. As a result of these combined stimulations, improvements in cognition could be observed even after an intervention of short duration, as we demonstrated in a previous paper [5] for motor coordination training. This hypothesis is also consistent with Diamond and Ling’s [23] statement that the effects of endurance exercise on cognition were more marked when supported by complex motor skills (see also Pesce [2], for a convergent point of view). 

### 4.1. Effects of the NW Training Program on Physical and Motor Functions

Results observed for the SWT confirmed that endurance capacity was enhanced by training in all the participants. Indeed, the distance covered significantly improved during the second half of the training program (S4W) by about 10% and finally by a similar amplitude after 8 weeks. Notably, progress was even larger among the responders (i.e., 26.5%), the number of which reached 64% during the second half of the training program. These results can be compared to those observed in our previous study [18], for the 6 min walking test (6WT), after 12 weeks of NW training: 90% of the participants were responders but their amplitude of progress was smaller than those observed in the present study (17%). Thus, it can be concluded that (i) the present training program strongly loaded cardiovascular capacities (as attested by the measure of training load during the sessions through HR), but (ii) a longer duration of the training intervention would be more effective in producing effects in (almost) all participants. Notably, progress in the distance covered was accompanied by a decrease in HRR, for which the number of responders was high, meaning that almost all participants were concerned by this benefit, mainly during the first 4 weeks of training, while progress in the distance covered was rather observed during the second 4-week period. This result suggested that training led to a deep cardiovascular effect (i.e., change in HRR), which could be a functional precursor of progress in distance covered during the SWT. 

Performance in the STS test, presumably reflecting muscular force, was also improved during the first 4 weeks of training and was then stabilized during the second 4-week period. Concerning motor functions, only coordination capacities were affected by NW training. In this test, the number of responders was high (>85%) and progress observed was large (24%) during the second 4 weeks and, finally, at the end of the training period. This result is fully consistent with those observed in the same test in our previous study [18]. 

NW training also improved coordination capacities, as attested by the results observed in the FSST. Indeed, progress was large in this domain (>24%), which is consistent with our previous study.

Notably, 8 weeks of training was not enough to improve the TUG at the preferred speed. This result is consistent with those observed in our previous study. Indeed, in this study, even after 12 weeks, progress was slight (12%). On the other, the lack of significant effect of NW training on equilibrium control (i.e., one-leg stance test) is more surprising. Indeed, in our previous study participants progressed by about 60% in this test after 12 weeks. Again, perhaps the program duration was too short to lead to progress in equilibrium control.

Finally, it can be concluded from these results that an 8-week training program of Nordic walking may be effective in improving endurance capacities, lower limb strength, and motor coordination. However, a caveat is in order concerning endurance capacities. Indeed, it appeared that only a small part of the group was significantly responsive after 8 weeks of training. Thus, regarding our theoretical hypotheses, this result suggests that even if NW requires a higher physical and muscular load than natural walking [11,22,45,46] and though significant progress was observed in physical and motor capacities after 8 weeks of training, a 12-week duration of training can be recommended to ensure that large benefits in physical and motor functions will be observed for almost all participants. 

### 4.2. Effects of NW Training on Cognitive Functions

Concerning cognition, we were expecting to confirm the results of our previous study [18], showing that NW training intervention led to significant progress in performance in different cognitive domains. Specifically, we expected to observe training-related benefits for inhibition and switching, together with information processing speed and visuospatial capacities. Beyond the facilitation role of aerobic effort through the release of neurotrophic factors [14], benefits for cognition were also expected to be related to the strong involvement of complex motor skills during NW training (as attested by the results observed for the FSST) [3,5,15,47]. Specifically, effects on inhibition were expected due to the demonstrated role of these cognitive processes in gait control [48,49]. Effects on switching could be observed because these processes were required during NW to change cognitive focus from one task to another that is, to orient oneself in the natural environment to control upright balance, to modify the length of the steps, to coordinate the two arms, or to use the biomechanical support of the sticks [50,51]. Effects on information processing speed were expected according to the results reported in previous studies on physical activity, in particular those requiring endurance effort [52]. Benefits on visuospatial capacities could result from the particular attention devoted to exercises requiring spatial orientation in natural environments during the present training program.

All these predictions were not fully confirmed by statistical results for the whole group of participants. Indeed, analyses carried out for all participants’ raw data only reported benefits on visuospatial capacities. This is not surprising because visuospatial capacities were strongly engaged in most exercises during the training period. Nevertheless, this result only partially aligned with those reported in our previous study [18] in which only 40% of the participants improved their visuospatial capacities, while there were more than 70% in the present study. In addition, in the present study, the amplitude of progress observed for all participants was large after 8 weeks of training (20–30%) and roughly similar to progress observed in Temprado et al.’s study [18] in the group of responders only. Notably, progress was mainly observed during the second 4 weeks of the training period. 

More surprising was the lack of significant effects observed for the other cognitive functions (i.e., inhibition, switching) and information processing speed when considering the whole group of participants. This was probably due to the small number of responders in these different cognitive domains. However, this is not to say that NW was ineffective in improving EF and information processing speed. Indeed, when considering the responders only, the amplitude of progress observed in the different tests after 8 weeks of training was rather large (i.e., >26% in TMT A and B and the different conditions of the CSWT). Concerning the theoretical hypotheses motivating the present study, these results suggest that NW has the potential to improve EF and, especially switching and inhibition, under the reserve that the training duration is long enough to generalize its effects in a majority of participants. Further studies should better explore the underlying mechanisms explaining the differences between responders and non-responders.

Another possible explanation for the lack of effects of NW training on EF in the majority of participants is that the effort intensity individually made by the participants over the program duration was too low [17,53]. Indeed, it is now well recognized that the intensity of aerobic training is one of the prominent moderators of brain plasticity and improvement of cognitive performance, through the release of neurotrophic factors in the blood. Though, on average, the training load falls within the prescribed range over the training period (60–80% of HRmax), it might be that individual intensity varied from one session to the other, thereby precluding strong effects in all the participants, as suggested by the results reported for the distance covered during the SWT. This hypothesis is in line with the results reported by Mortimer and colleagues [54], who showed that, when stratifying their group of participants into slow and fast walkers, fast walkers significantly improved their performance of EF compared to slow walkers. In the present study, our external observer reported that, though participants were encouraged to keep the intensity within the fixed range, the pace adopted by a number of them during some sessions was relatively slow to reach a more comfortable and habitual walking pace, in particular during the first 4 weeks training period. Combined with the short duration of the intervention, this could explain why effects on EF are not observed in a sufficient number of participants to result in statistically significant improvements.

## 5. Conclusions, Limitations, and Perspectives

The results suggest that an 8-week regimen of NW training is sufficient to give rise to progress in functionally important physical and motor functions that may help to preserve autonomy in older adults. On the other hand, to improve cognition in almost all participants, a 12-week training program would be presumably more effective, as suggested by our previous study [18]). 

In addition, our results suggest that benefits on visuospatial capacities are easy to obtain thanks to the inclusion of exercises requiring spatial orientation in natural environments. For the other cognitive functions, a combination of longer program duration (e.g., 12 weeks) and higher intensity of endurance effort could be recommended. However, since the benefits of NW for cognition could also result from the repetitive practice of complex movement (coordination, postural control, real-time adaptation of gait, etc.) together with cognitive tasks (e.g. spatial orientation [3,5,10,13,55]), the question arises of whether these cognitive–motor processes were sufficiently loaded during the NW intervention, despite the progress observed in the FSST. 

Accordingly, future studies should try to isolate the effects that come from aerobic effort and those triggered by repetitive practice of coordination and complex movements on cognitive performance during Nordic walking training; additionally, a larger sample size would enhance the generalizability of our findings. Notably, however, after the potential of NW training was demonstrated in a previous study [18], the present study was primarily designed as proof-of-concept research aiming to explore its time-dependent potential benefits. In this respect, our results showed promising outcomes, even if the small sample size and the lack of a control group can be considered serious limitations that prevent definitive and robust conclusions from being formulated. Finally, several research directions can be considered to extend the present study. First of all, it would be interesting to include longitudinal follow-up measures to evaluate the long-term efficacy and sustainability of the benefits observed from Nordic walking training. Indeed, it is crucial to explore the duration over which the improvements in cognitive, physical, and motor functions could be maintained post-intervention and to assess long-term adaptations among participants. This would make it possible to determine the dynamics of attenuation of the effects of training on physical, motor, and cognitive abilities. This question is important for establishing recommendations regarding alternating training and rest periods in older adults. However, such longitudinal studies are rarely performed, even though they would not only contribute significantly to our understanding of the impact of physical activity on the health and well-being of older adults but also potentially provide valuable insights for the development of recommendations about how to include NW training programs in everyday life in older adults. A second research direction concerns the comparison of the benefits of NW training programs with other activities. For example, although the clinical benefits of NW have been highlighted in numerous studies [13,56] and are lower than those of dance [19] or equivalent to those of tai chi intervention programs, the present study does not enable a comparison of benefits between NW and other forms of training. This will be performed in further studies.

## Figures and Tables

**Table 1:** Sample characteristics (M ± SD).

	M ± SD
Age (years)	71.1 ± 3.7
Gender	10 F – 4 M
Height (cm)	160.6 ± 6.5
Weight (kg)	68.4 ± 9.6
BMI	26.5 ± 3.7

**Table 2:** Attendance rates.

	F4W (12 SESSIONS = 100%)	S4W (12 SESSIONS = 100%)	T8W (24 SESSIONS = 100%)
NO. OF PARTICIPANTS	Mean No.; Mean %; (SD)	Mean No.; Mean %; (SD)	Mean No.; Mean %; (SD)
14	10.29; 85.71% (1.33)	9.86; 82.14% (1.61)	20.14; 83.93% (2.21)

Abbreviations: F4W, First 4 Weeks; S4W, Second 4 Weeks, T8W, Total 8 Weeks.

**Table 3:** Mean and maximum HR during the Nordic walking sessions.

	F4W			S4W			T8W		
NO. OF PARTICIPANTS	Mean HR	Mean HR Max	HR%	Mean HR	Mean HR Max	HR%	Mean HR	Mean HR Max	HR%
14	102.9	134	85%	103.9	118.7	75%	103.9	131.7	83%

Ann. Maximal HR was calculated using the formula from Tanaka et al. (2001) [26]; HR% was then calculated as (Mean HR max/Maximal HR) * 100. Abbreviations: F4W, First 4 Weeks; S4W, Second 4 Weeks, T8W, Total 8 Weeks.

**Table 4:** (A) The number of responders (N. %) expressed as a percentage of the whole group and their mean amplitude of progress (|Δ%|) expressed in absolute values for cognitive, motor, and physical outcomes over the three time points. (All deltas indicate an improvement in the performance of the various tests). (B) The amplitude of progress (Δ%) of all participants expressed in absolute values for each cognitive, motor, and physical test over the three time points. (All deltas indicate an improvement in the performance of the various tests.).

**(A)**						
	**F4W**		**S4W**		**T8W**	
PHYSICAL ASSESSMENTS						
TEST	N. (%)	|Δ%|	N. (%)	|Δ%|	N. (%)	|Δ%|
SWT—MAXIMAL HR	71	12.2	29	9.7	43	20.7
SWT-HRR	93	18.1	36	7.1	93	23.9
SWT—DISTANCE COVERED	14	15.3	64	26.5	36	19.1
STS	100	11.7	64	14.4	86	24.3
MOTOR TESTS						
FSST	57	16.7	93	20.7	86	33.9
BALANCE	79	373.5	36	179.8	64	180.5
TUG	36	8.4	50	5.7	29	10.5
COGNITIVE TESTS						
REY TEST TIME	71	30.2	71	16.4	79	41.7
REY TEST SCORES	50	2.5	0	0	21	2.5
TMT A TIME	57	15.9	71	14.3	64	27.8
TMT A N. OF ERRORS	36	95	7	66.6	29	75
TMT B TIME	50	22.6	50	9.6	36	31
TMT B N. OF ERRORS	50	95.8	14	100	50	100
CWST RT—C	57	8.5	43	6.5	57	14.7
CWST RT—I	43	5.5	64	6.9	57	12.2
CWST RT—N	57	18.8	57	8.7	64	26
CWST N. OF ERRORS—C						
CWST N. OF ERRORS—I	36	90	36	85	36	90
CWST N. OF ERRORS—N	14	100	21	100	14	100
MOCA	43	3.9	57	9.7	50	13.9
**(B)**						
**TEST**	**F4W**		**S8W**		**T8W**	
PHYSICAL ASSESSMENTS						
SWT—MAXIMAL HR	3.6		6.8		2.6	
SWT-HRR	15.7		1.8		14.9	
SWT—DISTANCE COVERED	4.7		14.2		9.5	
STS	12.9		4.1		16.7	
MOTOR TESTS						
FSST	0.8		23.6		25.7	
BALANCE	208.1		7.1		59.3	
TUG	5.1		0.2		4.8	
COGNITIVE TESTS						
REY TEST TIME	14.3		4.9		21.4	
REY TEST SCORES	11.6		5.6		5.0	
TMT A TIME	4.1		5.8		11.7	
TMT A N. OF ERRORS	40 *		0 *		40 *	
TMT B TIME	0.2		8.5		2.8	
TMT B N. OF ERRORS	76 *		40 *		116 *	
CWST RT—C	3.7		2		2.4	
CWST RT—I	0.5		3.5		4.8	
CWST RT—N	6.7		2		5.2	
CWST N. OF ERRORS—C	0 *		32 *		32 *	
CWST N. OF ERRORS—I	8 *		4 *		12 *	
CWST N. OF ERRORS—N	8 *		32 *		24 *	
MOCA	1.6		2.7		4.1	

(A) Abbreviations: F4W, First 4 Weeks; S4W, Second 4 Weeks, T8W, Total 8 Weeks; SWT, Shuttle Walking Test; HR, Heart Rate; HRR, Heart Rate Rest; STS, Sit-To-Stand; FSST, Four-Square Stepping Test; TUG, Timed Up and Go; TMT A and B, Trail Making Test A and B; CWST, Color–Word Stroop Test; RT, Response Time; C, Congruent; I, Incongruent; N, Neutral; MoCA, Montreal Cognitive Assessment. (B) * refers to the difference in raw scores (i.e., F4W = INT-score − PRE-score; S8W = POST-score − INT-score; T8W = POST-score − PRE-score) between the time points, expressed as a percentage. Abbreviations: F4W, First 4 Weeks; S4W, Second 4 Weeks, T8W, Total 8 Weeks; SWT, Shuttle Walking Test; HR, Heart Rate; HRR, Heart Rate Rest; STS, Sit-To-Stand; FSST, Four-Square Stepping Test; TUG, Timed Up and Go; TMT A and B, Trail Making Test A and B; CWST, Color–Word Stroop Test; RT, Response Time; C, Congruent; I, Incongruent; N, Neutral; MoCA, Montreal Cognitive Assessment.

## Data Availability

Data are available on demand to the first author.
